# Gut Bacterial Species Distinctively Impact Host Purine Metabolites during Aging in *Drosophila*

**DOI:** 10.1016/j.isci.2020.101477

**Published:** 2020-09-10

**Authors:** Toshitaka Yamauchi, Ayano Oi, Hina Kosakamoto, Yoriko Akuzawa-Tokita, Takumi Murakami, Hiroshi Mori, Masayuki Miura, Fumiaki Obata

**Affiliations:** 1 Department of Genetics, Graduate School of Pharmaceutical Sciences, the University of Tokyo, Bunkyo-ku, Tokyo 113-0033, Japan; 2 Department of Informatics, National Institute of Genetics, Mishima, Shizuoka 411-8540, Japan

**Keywords:** Biological Sciences, Molecular Biology, Immunology, Microbiome

## Abstract

Gut microbiota impacts the host metabolome and affects its health span. How bacterial species in the gut influence age-dependent metabolic alteration has not been elucidated. Here we show in *Drosophila melanogaster* that allantoin, an end product of purine metabolism, is increased during aging in a microbiota-dependent manner. Allantoin levels are low in young flies but are commonly elevated upon lifespan-shortening dietary manipulations such as high-purine, high-sugar, or high-yeast feeding. Removing *Acetobacter persici* in the *Drosophila* microbiome attenuated age-dependent allantoin increase. Mono-association with *A. persici*, but not with *Lactobacillus plantarum*, increased allantoin in aged flies. *A. persici* increased allantoin via activation of innate immune signaling IMD pathway in the renal tubules. On the other hand, analysis of bacteria-conditioned diets revealed that *L. plantarum* can decrease allantoin by reducing purines in the diet. These data together demonstrate species-specific regulations of host purine levels by the gut microbiome.

## Highlights

• Gut bacterial species regulate the age-dependent metabolic shift in *Drosophila*
• *Acetobacter persici* increases purine levels via IMD activation in the renal tubules
• *Lactobacillus plantarum* decreases the purine levels in the *Drosophila* diet
• Diet- and bacteria-dependent elevation of purine levels may shorten the lifespan

## Graphical Abstract

**Figure:**
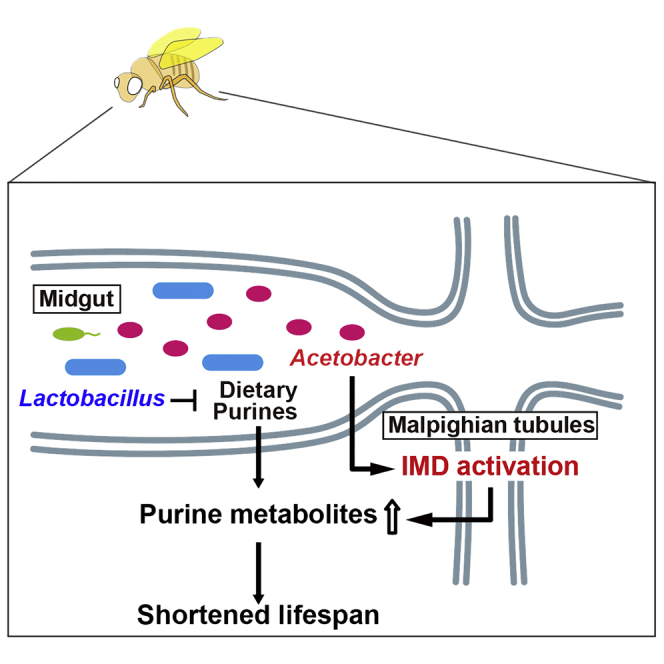

## Introduction

Commensal bacteria have a profound effect on host health. Gut microbiota can influence the host metabolome, as they modulate dietary components and provide some metabolites directly to the host (Tang et al., 2019; Visconti et al., 2019). It is also possible that some bacterial cues stimulate specific metabolic pathways in the host. However, the detailed connection between the microbiome and the host metabolome, particularly in the context of aging, is only beginning to be identified.

*Drosophila melanogaster* is a powerful model for the mechanistic elucidation of host-microbiome interaction during aging. The advantages of *Drosophila*, with its abundant genetic tools, include the relatively short lifespan and a small number of indigenous bacterial genera, predominantly *Lactobacillus* and *Acetobacter* (Erkosar et al., 2013; Miguel-Aliaga et al., 2018). The simple bacterial communities, nevertheless, can influence host aging, during which the gut microbiome becomes dysbiotic (Clark et al., 2015; Guo et al., 2014). Several metabolites produced by the *Drosophila* microbiota are known to limit the host lifespan. For instance, some bacterial species, such as *Lactobacillus brevis* or *Gluconobacter morbifer*, produce uracil and elicit intestinal damage (Lee et al., 2013). An expansion of *Lactobacillus plantarum* in the gut of null mutant of the immune regulator *PGRP-SD* shortens the lifespan through lactate (Iatsenko et al., 2018). Although many bacteria-derived metabolites that affect health span have been identified, bacteria-dependent reduction of nutrient in the host diet remains unexplored. Besides, there are still many questions on how microbiota regulate the host metabolic pathways during aging.

Here, we performed metabolome analyses to identify how gut microbiota influence the age-related metabolic trajectory in *Drosophila*. Allantoin was found to be increased during aging in flies with normal microbiome but not with depleted *Acetobacter persici*. We also found that *L. plantarum* reduced purine levels from the fly diet. In this study, we revealed how dietary and bacterial factors regulate allantoin as a marker of total purine levels in the body.

## Results

### Microbiome Affects Age-Dependent Metabolic Shift

**Figure 1:**
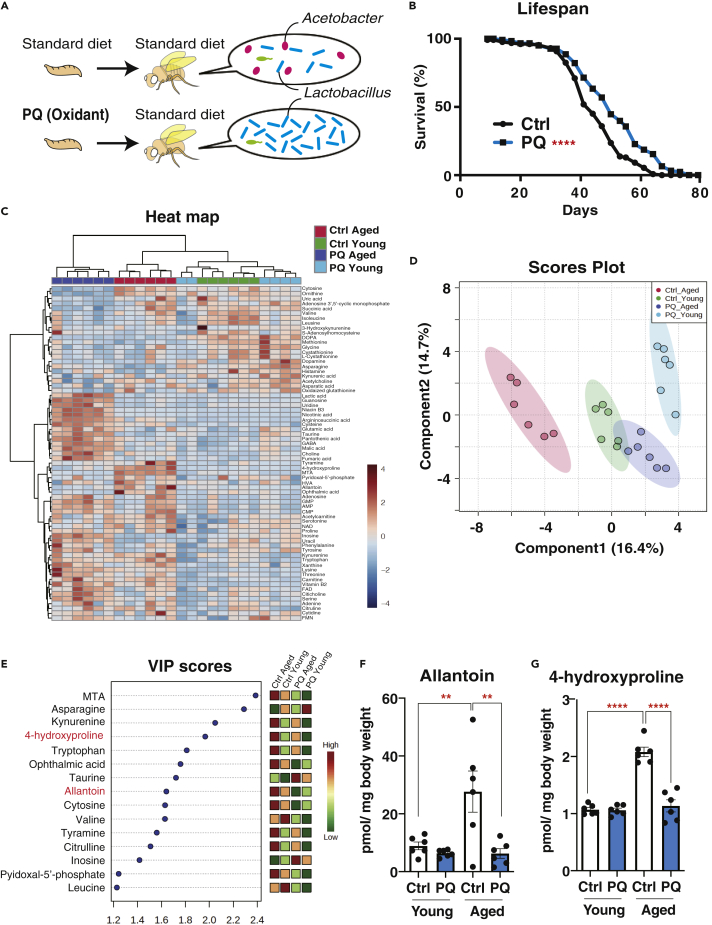
The Altered Microbiome by Oxidant Alters Age-Related Metabolic Trajectory (A) Overview of phenotypes of the paraquat (oxidant)-experienced flies. (B) Lifespan of control (Ctrl) or paraquat (PQ)-exposed *w^Dah^* male flies. *n* = 131 (Ctrl), n = 123 (PQ). Log rank test, p < 0.0001. (C–E) Whole body metabolome in young (1-week old) or aged (5-week old) *w^Dah^* male flies with or without PQ treatment during larval stage. n = 6. Heatmap (C), PLS-DA analysis (D), and Variable Importance in Projection scores (E) are shown. (F and G) Quantification of allantoin (F) and 4-hydoxyproline (G) by LC-MS/MS in whole body of young or aged *w^Dah^* male flies with or without PQ treatment during larval stage. Data are represented as mean and SEM. n = 6. Statistics: one-way ANOVA with Sidak's multiple comparison test. ∗*∗*p < 0.01. ∗∗∗∗p < 0.0001.

A previous study showed that low-dose oxidants such as paraquat during development selectively deplete *Acetobacteraceae* and expand *Lactobacillaceae* (Obata et al., 2018). This altered microbiome suppresses age-related immune activation and intestinal dysfunction, leading to lifespan extension (Figures 1A and 1B). To identify how the microbiome remodeling affects the host metabolome during aging, we quantified whole-body metabolites in young or aged male flies with or without oxidant (paraquat) experience. Liquid chromatography/tandem mass spectrometry (LC-MS/MS) was used to quantify metabolites in the whole-body samples from 1-week-old versus 5-week-old flies; at that time the two lifespan curves did not differ significantly (Figure 1B). The analysis enabled us to quantify the 69 metabolites in this setting. A heatmap analysis revealed that the metabolome of paraquat-experienced young flies was not separated well from that of control flies (Figure 1C, green versus light blue). When the flies were aged, in contrast, the cluster between the two conditions became distinct (Figure 1C, red versus blue). Furthermore, a partial least squares discriminant analysis (PLS-DA) showed that the metabolome of control flies strongly shifted during aging along with the component 1 axis, whereas that of paraquat-experienced flies did not (Figure 1D). These data implied that the gut microbiome influenced the flies' “age-dependent” metabolic trajectory.

Among the top 15 metabolites in Variable Importance in Projection scores, which are used in a PLS model to estimate a possible variable for the division among conditions (Figure 1E), we found that allantoin and 4-hydroxyproline were robustly increased during aging in all tested “control” strains, *w*^*Dahomey*^, *Canton S,* and *w*^*iso31*^ (Figures 1F, 1G, S1A, and S1B). The increases of these two metabolites during aging were suppressed by early-life paraquat exposure (Figures 1F and 1G). Thus, these metabolites were regulated in a bacteria-dependent manner. Neither allantoin nor 4-hydroxyproline feeding shortened the lifespan, suggesting that these metabolites *per se* were not detrimental for flies (Figures S1C and S1D). Therefore, we decided to understand the mechanism regarding how these metabolites were increased during aging.

### Allantoin Is Synthesized upon Increase of the Total Purine Levels

**Figure 2:**
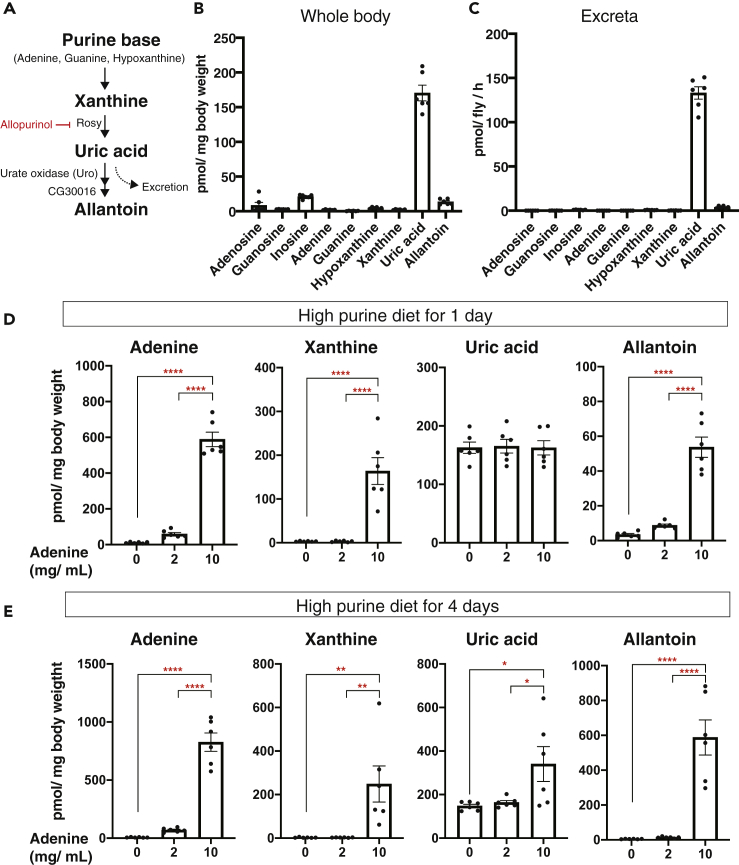
Allantoin Is Produced upon Excessive Purines (A) Purine metabolism in *Drosophila melanogaster*. (B and C) Quantification of purine metabolites by LC-MS/MS in whole body (B) or excreta (C) of young (1-week-old) *w*^*Dah*^ male flies. n = 6. (D and E) Quantification of adenine, xanthine, uric acid, and allantoin by LC-MS/MS in whole body of young (2-week-old) *w*^*Dah*^ male flies fed with a high-purine diet for 1 day (D) or 4 days (E). n = 6. Data are represented as mean and SEM. Statistics: one-way ANOVA with Tukey's multiple comparison test. ∗p < 0.05. ∗∗p < 0.01. ∗∗∗∗p < 0.0001.

Allantoin is an end product of purine degradation pathway in *Drosophila* (Figure 2A). In humans, excess purine bodies are metabolized into uric acid to excrete them from the body. Many other animals, including mice and flies, have functional urate oxidase (Uro), which enables further degradation of uric acid into allantoin. We first hypothesized that the age-dependent accumulation of allantoin might be due to the dysfunction of the excretion process. We conducted an “excretion assay” using blue-dye food (Figure S2A, Shell et al., 2018). Unexpectedly, the total capacity of excretion did not decrease during aging (Figure S2B). We quantified allantoin levels in collected excreta using LC-MS/MS to test whether allantoin excretion was specifically defective in the aged flies. The amount of allantoin in excreta was not decreased, rather it was slightly increased (Figure S2C), suggesting that old flies were capable of, more or less, excreting the metabolite. Theoretically, an increase of food intake can upregulate allantoin levels. However, the capillary feeder assay (Ja et al., 2007) showed that food intake was decreased during aging rather than increased (Figure S2D).

Next, we tested whether allantoin synthesis was upregulated in the aged flies. *Drosophila Uro* is predominantly expressed in the Malpighian tubules, the fly counterpart of renal tubules (flybase.org). Gene expression of allantoin synthase *Uro* as well as its up- and downstream enzymes, *Rosy* and *CG30016,* was not increased, at least transcriptionally, during aging in the Malpighian tubules (Figures 2A and S2E). Thus, the upregulation of total purine levels, or its flux, is likely to be a cause of the age-related increase of allantoin. Interestingly, when we quantified each purine metabolite in young male flies, we noticed that the basal level of allantoin was relatively low compared with that of uric acid (Figure 2B). It is believed that a majority of excess purines, or nitrogen generally, are excreted in the form of allantoin. However, this pattern was also the case in the excreta metabolites, suggesting that uric acid, not allantoin, is excreted despite flies having functional Uro (Figure 2C).

To characterize the allantoin metabolism in flies, we fed male flies with allopurinol, an inhibitor of xanthine oxidase used for treating hyperuricemia in humans (Figure 2A). Both uric acid and allantoin in the whole body were decreased, whereas xanthine was increased (Figure S3A). On the other hand, feeding young male flies with a high-adenine diet led to an increased allantoin, uric acid, and xanthine in a dose- and duration-dependent manner (Figures 2D and 2E). The allantoin level in the excreta was also elevated upon adenine feeding, whereas uric acid was rather decreased for an unknown reason (Figure S3B).

**Figure 3:**
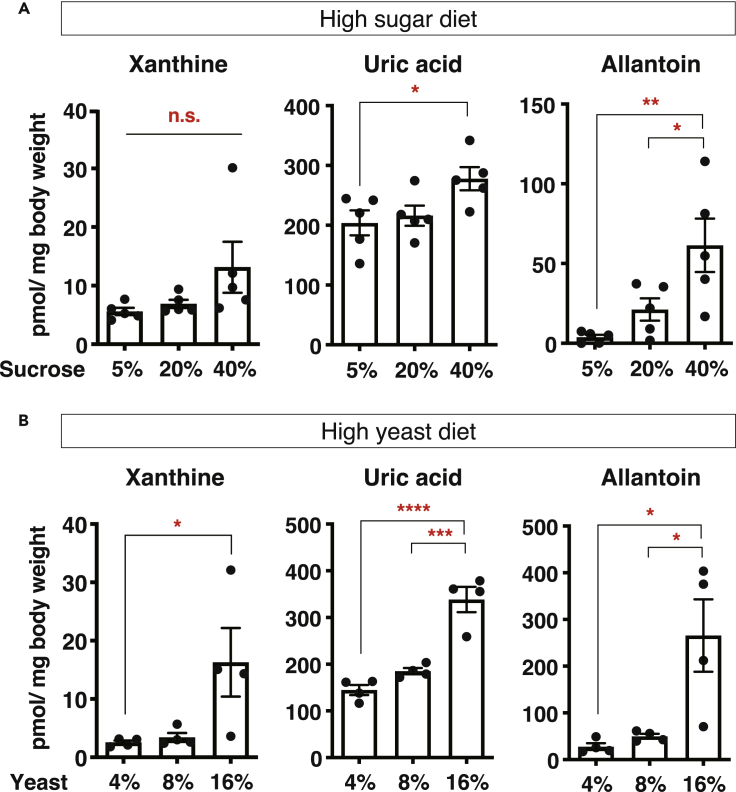
High-Sugar and High-Yeast Diets Increase Allantoin (A and B) Quantification of xanthine, uric acid, and allantoin by LC-MS/MS in whole body of young (1-week-old) *w*^*Dah*^ male flies fed with a high-sugar diet (A, n = 5) or a high-yeast diet (B, n = 4) for 3 days. Data are represented as mean and SEM. Statistics: one-way ANOVA with Tukey's multiple comparison test. ∗p < 0.05. ∗∗p < 0.01. ∗∗∗p < 0.001. ∗∗∗*∗*p < 0.0001.

The increased allantoin level by adenine feeding was correlated with shortened lifespan (Figure S3C), consistent with the previous report (van Dam et al., 2020). Other dietary nutrients, such as sugars and amino acids, can provide with substrates for purine biosynthesis. A high-sugar diet causes early mortality due to the increased uric acid production and concomitant renal stones in flies (van Dam et al., 2020). High-yeast feeding also induces uric acid accumulation and shortens the lifespan of *Uro* knockdown flies (Lang et al., 2019). As expected, allantoin, as well as xanthine and uric acid, was increased in whole body by both dietary manipulations (Figures 3A and 3B). Taken together, the allantoin level in flies is commonly elevated upon these lifespan-shortening dietary conditions. During aging, adenosine and xanthine, as well as allantoin, were increased in male flies at 5 weeks of age, although adenine and uric acid were not (Figure S3D). The increased allantoin in aged flies might be due to the increased total purine levels. It is possible that uric acid levels are maintained during aging because allantoin synthesis can buffer the increased purine metabolite levels to a certain extent. We assume that uric acid can also elevate during aging when the total purine levels are beyond the animals' capacity to handle.

### *Lactobacillus plantarum* Decreases Dietary Purines

**Figure 4:**
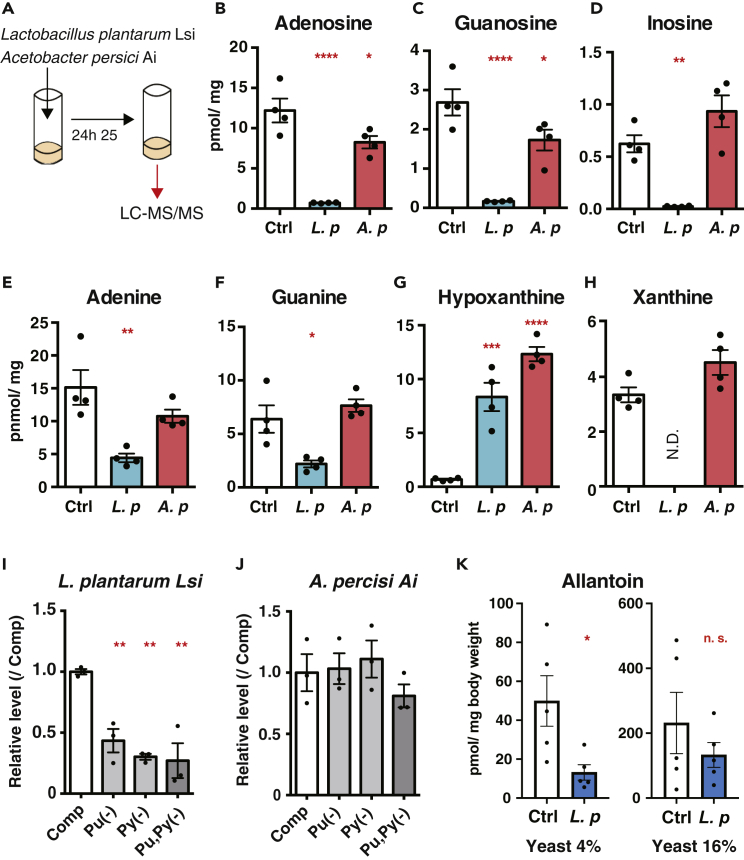
*Lactobacillus Plantarum* Decreases Purine Metabolites (A) Experimental scheme of bacterial conditioning assay. (B–H) Quantification of (B) adenosine, (C) guanosine, (D) inosine, (E) adenine, (F) guanine, (G) hypoxanthine, and (H) xanthine by LC-MS/MS in bacterial-conditioned diet with isolated strains. Ctrl, bacterial culture medium; *L. p*, *Lactobacillus plantarum* Lsi; *A. p*, *Acetobacter persici* Ai. n = 4. (I and J) Bacterial growth of *L. plantarum* Lsi (I) and *A. persici* Ai (J) in purine- or pyrimidine-depleted medium during 21 h of incubation. Relative absorbance (600 nm) to complete medium (Comp) are shown. n = 3. (K) Quantification of allantoin by LC-MS/MS in whole body of young (1-week-old) *w*^*Dah*^ male flies fed with bacterial pre-conditioned diet. n = 5. Data are represented as mean and SEM. Statistics: one-way ANOVA with Dunnett's multiple comparison test (B–J) or a two-tailed Student t test (K). ∗p < 0.05, ∗∗p < 0.01, ∗∗∗p < 0.001, ∗∗∗∗p < 0.0001.

Purines are either synthesized in cells or ingested as nutrients. Considering that the microbiome possesses its unique metabolic pathways, commensal bacterial species can modulate the purine levels in the *Drosophila* diet. To test this possibility, we conducted a bacterial-conditioning assay (Figure 4A). Bacterial isolates were added to standard fly diets and incubated at 25°C for 24 hours. The composition of this conditioned diet was assessed by LC-MS/MS. In this experiment, we used *Lactobacillus plantarum* Lsi and *Acetobacter persici* Ai, both of which were previously isolated in our laboratory (see Methods). Given that removing *Acetobacteraceae* by paraquat reduces allantoin in aged flies (Figure 1F), it was expected that *A. persici* Ai would produce purines. However, *A. persici* Ai did not increase purines in the diet, except for hypoxanthine; but rather, mildly decreased purine nucleosides, adenosine and guanosine (Figures 4B–4H).

Surprisingly, we observed a sharp reduction of all three purine nucleosides by conditioning with *L. plantarum* Lsi (Figures 4B–4D). Not only purine nucleosides but also purine bases (adenine and guanine), xanthine, pyrimidine nucleosides (cytidine, thymidine, and uridine), and uracil were reduced in the conditioned diet with *L. plantarum* Lsi, whereas hypoxanthine, cytosine, and thymine were not (Figures 4E–4H and S4A–S4F). Allantoin was not detected in any of the conditioned diets. Next, we examined the specificity of the phenotype by testing other *Acetobacter* species, such as *Acetobacter aceti*, *Acetobacter tropicalis,* and *Acetobacter pasteurianus* in a small scale using a 1.5-mL tube (Figure S4G); none of them decreased adenosine (Figure S4H). In contrast, *Lactobacillus* *brevis*, but not *Lactobacillus acidophilus* and *Lactobacillus murinus*, decreased dietary adenosine (Figure S4I), which suggested that the ability of adenosine reduction is specific to some *Lactobacillus* species.

To understand what determines the capacity to decrease the purine levels, we performed a comparative analysis of the four *Lactobacilli* genomes. *L.* *brevis* lacked the majority of the genes involved in *de novo* purine synthesis, consistent with the fact that this bacterium needed to utilize adenosine in the fly diet. Unexpectedly, not only *L. acidophilus* and *L. murinus* but also *L. plantarum* Lsi possess genes for the *de novo* purine synthesis pathway. We also inspected genes encoding transporters to ask whether specific expression of the transporters explains the difference in purine metabolism. *L. plantarum* Lsi and *L. brevis* had homologs of the purine-cytosine transporter *codB*, whereas the other two strains did not. In contrast, there is a gene for NupC/NupG family nucleoside transporter in the genome of *L. murinus*, *L. brevis*, and *L. plantarum,* whereas *L. acidophilus* possessed a gene set encoding BmpA-NupABC, an ATP-binding cassette transporter for nucleosides. The reason why some but not all *Lactobacillus* species reduced environmental nucleosides was not obvious from the genome comparison.

We noticed that the difference in the ability to decrease purine nucleosides among *Lactobacillus* species was correlated with the speed of bacterial growth (Figure S4J). This observation led to the assumption that *L. plantarum* Lsi could utilize extracellular nucleic acids for rapid growth. To test this hypothesis, we analyzed the bacterial growth on a chemically defined medium (see Methods). The medium contains the nucleosides inosine and uridine as sole purine and pyrimidine sources, respectively. The growth of *L. plantarum* Lsi was suppressed, if not abolished, upon either inosine or uridine depletion (Figure 4I), whereas that of *A. persici* was not affected at all (Figure 4J). These data implied that the gut bacteria *L. plantarum* decreased the purine metabolites of the fly diet by using them for promoting bacterial growth.

To test whether this bacterial metabolism can influence fly metabolome, we fed young flies with *L. plantarum* Lsi-conditioned diet. The bacterial conditioning of the low-yeast diet, but not the high-yeast diet, led to the decrease of allantoin levels in flies (Figure 4K). Therefore, commensal bacterium *L. plantarum* could reduce host purine levels by direct modulation of the host's diet, although this is dependent on dietary condition.

On the other hand, the bacterial-conditioned diet with *A. persici* Ai had a significantly large amount of 4-hydroxyproline (Figure S4K). Given that *Acetobacteraceae* is increased during aging (Guo et al., 2014; Obata et al., 2018), the age-dependent increase of 4-hydroxyproline might be attributable to an elevated amount of direct provision by *A. persici*.

### *Acetobacter persici* Increases Allantoin via IMD Activation

**Figure 5:**
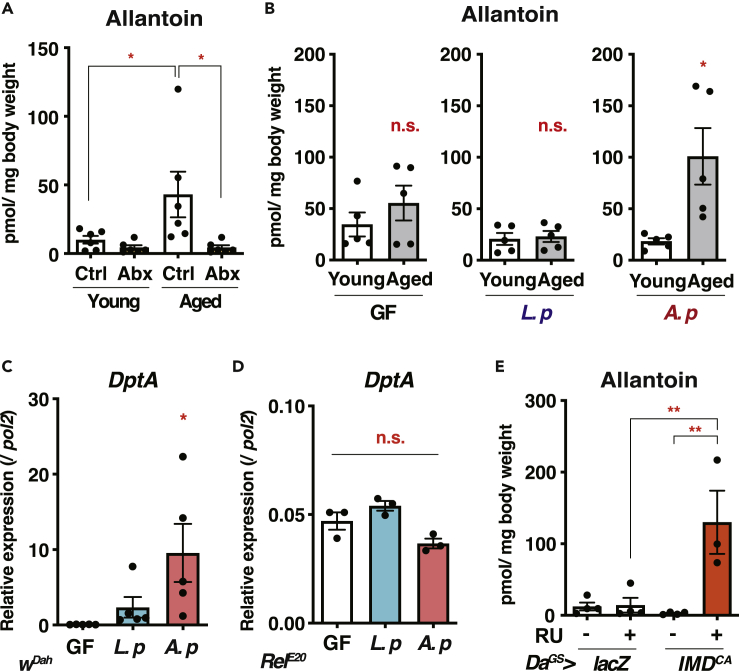
*Acetobacter Persici* Activates IMD and Increases Allantoin during Aging (A) Quantification of allantoin by LC-MS/MS in whole body of young (2-week-old) or aged (6-week old) *w*^*Dah*^ male flies with or without antibiotic treatment. n = 6. (B) Quantification of allantoin by LC-MS/MS in whole body of young (1-week-old) or aged (5-week-old) *w*^*Dah*^ male flies mono-associated with bacterial strains. GF, germ-free; *L.p*, *Lactobacillus plantarum* Lsi; *A.p*, *Acetobacter persici* Ai. n = 5. (C and D) Quantitative RT-PCR analysis of *Diptericin A* (*DptA*) in whole body of young (1-week-old) male flies mono-associated with the bacterial strains. n = 5 for *w*^*Dah*^ flies (C) or n = 3 for *Relish* mutant flies (*Rel*^*E20*^) (D). (E) Quantification of allantoin by LC-MS/MS in whole body of young (2-week-old) male flies fed with antibiotics. Either *lacZ* or constitutive active form of IMD (*IMD*^*CA*^) was overexpressed ubiquitously by *Da*^*GS*^. RU486 is an inducer of GeneSwitch. n = 3−4. Data are represented as mean and SEM. Statistics: one-way ANOVA with Sidak's multiple comparison test (A and E), Dunnett's multiple comparison test (C and D), or a two-tailed Student t test (B). ∗p < 0.05, ∗∗p < 0.01.

The fact that *L. plantarum* decreases dietary purines suggested that abundant colonization of this bacterium in the paraquat-experienced flies (Figure 1A) might prevent the age-related increase of allantoin. If this is the case, removing this bacterium should result in increased allantoin during aging. Unexpectedly, the elimination of all bacterial species, including *L. plantarum*, by antibiotics suppressed the increase of allantoin (Figure 5A). These data therefore suggested that depletion of *A. persici* suppressed the age-related allantoin elevation. To test whether *A. persici* Ai was sufficient for the phenotype, we performed the gnotobiotic experiment. As we expected, *A. persici* Ai mono-association, but neither germ-free nor *L. plantarum* Lsi mono-association, increased the allantoin level during aging (Figure 5B). These data suggested that *A. persici* Ai was responsible for this phenotype. *L. plantarum* mono-associated flies showed a tendency of low allantoin levels compared with the germ-free flies, implying that the bacterium can contribute to better handling at purine levels during aging.

*Drosophila* has two innate immune signaling cascades, immune deficiency (IMD) and Toll pathways, the counterparts of mammalian tumor necrosis factor receptor and Toll-like receptor pathways, respectively. The IMD pathway is known to be hyperactivated during aging, which can be attenuated by removing microbiota (Clark et al., 2015; Guo et al., 2014; Obata et al., 2018). In our laboratory condition, aging expands the ratio of *Acetobacteraceae* to *Lactobacillaceae*, at least in *w*^*iso31*^ male flies (Figure S5A). Male flies with *A. persici* Ai mono-association showed higher levels of *Diptericin A* (*DptA*) expression, which is one of the readouts for IMD activation (Figure 5C), compared with germ-free or *L. plantarum* Lsi mono-associated flies. This is interesting given that IMD pathway could be activated by DAP-type peptidoglycan found in both gram-negative *A. persici* and gram-positive *Lactobacillus* spp. (Broderick and Lemaitre, 2012). A null mutation for the IMD-regulated transcription factor *Relish* abolished the induction of *DptA* by *A. persici* Ai (Figure 5D). In contrast, *A. persici* Ai did not upregulate a Toll readout *Drosomycin* (*Drs*) (Figure S5B). These data suggested that *A. persici* Ai is a potent activator of the IMD pathway.

To test straightforwardly whether IMD activation was sufficient for the age-dependent increase of allantoin levels, we overexpressed the constitutive active form of IMD (*IMD*^*CA*^) in young male flies under antibiotic treatment. Ubiquitous expression of *IMD*^*CA*^ by a drug-inducible GeneSwitch driver (*Da*^*GS*^) triggered a sharp increase of allantoin (Figure 5E).

### IMD Activation in the Renal Tubules Increases Allantoin in Aged Flies

**Figure 6:**
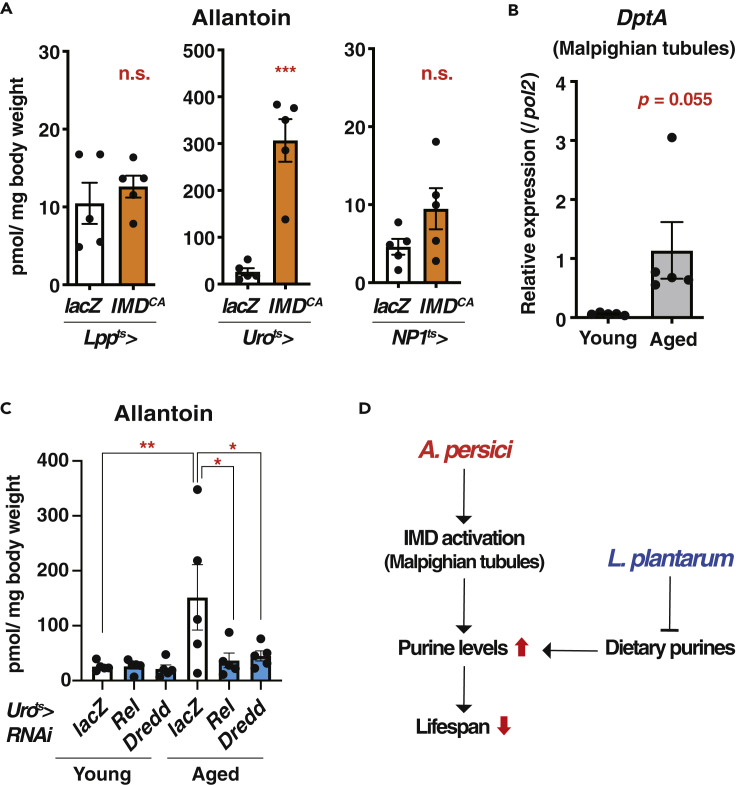
IMD Activation in the Renal Tubules Increases Allantoin (A) Quantification of allantoin by LC-MS/MS in whole body of young (2-week-old) male flies fed with antibiotics. Constitutive active form of IMD (*IMD*^*CA*^) is overexpressed using tissue-specific drivers (*Lpp-Gal4, tub-Gal80*^*ts*^: fat body, *Uro-Gal4, tub-Gal80*^*ts*^: Malpighian tubules, *NP1-Gal4, tub-Gal80*^*ts*^: gut). n = 5. (B) Quantitative RT-PCR analysis of *Diptericin A* (*DptA*) in the Malpighian tubules of young (1-week-old) and aged (6-week-old) *w*^*Dah*^ male flies. n = 5. (C) Quantification of allantoin by LC-MS/MS in whole body of young (1-week-old) and aged (4-week-old) male flies with *lacZ*, *Relish,* or *Dredd*-RNAi in the Malpighian tubules. n = 5. (D) Proposed model. Data are represented as mean and SEM. Statistics: a two-tailed Student t test (A and B) or one-way ANOVA with Sidak's multiple comparison test (C). ∗p < 0.05, ∗∗p < 0.01, ∗∗∗p < 0.001.

IMD pathway is activated predominantly via microbiota-produced peptidoglycan (PGN). Monomeric PGN can activate systemic IMD signaling (Charroux et al., 2018; Myllymäki et al., 2014). Interestingly, IMD activation in the Malpighian tubules, but not in the gut or fat body, increased allantoin (Figures 6A and S5C) and also tended to increase adenine and uric acid (Figure S5D). We confirmed that IMD activation was up-regulated in the aged Malpighian tubules (Figure 6B). Consistent with the phenotypes in aged flies (Figures S2D and S2E), neither gene expressions of allantoin synthesis enzymes nor food intake were increased by IMD activation in the Malpighian tubules (Figures S5E and S5F). The increase of allantoin in Malpighian tubule-specific activation of IMD pathway was correlated with a shortened lifespan (Figure S5G).

When *Relish* was knocked down in the Malpighian tubules, the age-dependent increase of allantoin was suppressed (Figure 6C). We confirmed the phenotype by knocking down *dredd*, a component of IMD pathway (Figure 6C), indicating the requirement of IMD activation in the Malpighian tubules for the increased allantoin during aging. Taken together, the gut microbial species *A. persici* and *L. plantarum* can influence the age-dependent metabolic trajectory of the purine metabolites, which may contribute to shortening the hosts' lifespan (Figure 6D).

## Discussion

Aging impacts metabolic alteration, which is influenced by gut microbiota. Despite the accumulation of descriptive omics data, our mechanistic understanding of age-dependent shift of the metabolome is still in its infancy. This study identified two metabolites, allantoin and 4-hydroxyproline, which were increased during aging in the gut microbiota-dependent manner. The bacterial-conditioning assay demonstrated that 4-hydroxyproline was directly produced by *A. persici* Ai, suggesting that the increase of 4-hydroxyproline is a signature of the expansion of this bacterium during aging. On the other hand, allantoin and many other purine metabolites were not directly produced by any bacterial species. Instead, the immuno-stimulatory capacity of *A. persici* accounts for the age-related increase of allantoin, likely through accelerated purine biosynthesis by the host. Interestingly, a rat model of hyperuricemia-induced nephropathy showed increased levels of uric acid and 4-hydroxyproline, a phenotype similar to our aged *Drosophila* (Pan et al., 2019).

Allantoin is identified as a potential caloric restriction mimetic, and feeding allantoin extends the lifespan in *C. elegans* (Calvert et al., 2016). We noticed that allantoin feeding slightly increased the fly lifespan. However, the elevation of endogenous allantoin by manipulation of dietary sugar, yeast, or purine was correlated with a shortened lifespan. The increase of pro-longevity allantoin can be an adaptive response to aging, or the increase serves simply as a marker for increased total purine levels. Some purines and uric acid levels are also increased in conditions with high allantoin levels, and uric acid accumulation is a cause of shortened lifespan by high-purine diets (van Dam et al., 2020; Lang et al., 2019). It might be interesting to test whether *Uro* overexpression can extend lifespan. Intriguingly, high-sugar diet induces dehydration, and the shortened lifespan of flies with the diet is fully restored by water supplementation (van Dam et al., 2020). High-yeast diet can also induce dehydration, implying that water loss is the common mechanism to increase purine levels by these dietary conditions (Ja et al., 2009). It is also noteworthy that dehydration stress (by decreased environmental humidity) activates innate immunity in the Malpighian tubules (Zheng et al., 2018). Whether the deleterious effect of renal immune activation (*Uro*^*ts*^ *> IMD*^*CA*^) on lifespan can be attenuated through water supplementation is worth testing.

Accumulating evidence suggests that chronic activation of inflammatory response is a key driver of aging. In *Drosophila*, systemic or intestinal IMD activation via commensal bacteria is believed to limit the lifespan (Clark et al., 2015; Guo et al., 2014). However, the mechanism by which IMD pathway shortens organismal lifespan is not fully understood. This study implies that the altered purine metabolism is one of the downstream events induced by the systemic IMD pathway. Allantoin is reported to be a biomarker of inflammation in a mouse model of inflammatory bowel diseases (Dryland et al., 2008). In humans, an age-related increase in serum uric acid levels has been widely observed (Dalbeth et al., 2016; Kuo et al., 2015; Kuzuya et al., 2002). Elevated uric acid is associated with many pathologies, including systemic inflammation (Lyngdoh et al., 2011) and mortality (Meisinger et al., 2008). The gut microbiota from patients with gout is significantly different from that of healthy humans (Guo et al., 2016). It is interesting that in goslings, the gut microbiota-derived lipopolysaccharide increased the risk of visceral gout (Xi et al., 2019). Therefore, the gut microbiota-dependent activation of innate immunity might be a general driver of hyperuricemia pathologies and organismal aging in mammals.

The fact that allantoin is increased by IMD activation in the Malpighian tubules implied that tissue- and bacteria-specific mechanisms of immune response impact on the purine metabolism. However, the detail of mechanism by which IMD pathway regulates purine metabolism is not elucidated. Considering that IMD activation does not directly upregulate expression levels of allantoin synthesis enzymes, it might regulate purine synthetic pathways. Purines can be synthesized *de novo* from glucose and amino acids through the pentose phosphate pathway (PPP). It is reported that glycolytic activity was attenuated during aging in *Drosophila* (Ma et al., 2018), potentially increasing glucose flux to PPP. Alternatively, age-dependent acceleration of protein catabolism, possibly via immune activation, can produce free amino acids, leading to an increased purine synthesis to excrete excess nitrogen. This assumption, however, is not supported well by the fact that IMD activation in the gut or fat body, the central metabolic organs, did not increase allantoin levels. In mammals, extracellular adenosine is known to be massively increased during inflammatory conditions via ATP breakdown (Antonioli et al., 2019). How IMD activity in the Malpighian tubules leads to allantoin accumulation is to be investigated in future studies.

Many intrinsic and extrinsic factors, such as age, genotype, or diet, contribute to shaping the bacterial communities (Claesson et al., 2011; Wan et al., 2019). Imbalanced bacterial communities (dysbiosis) compromise health span. Either the direct provision of beneficial bacterium (probiotics) or dietary intervention to increase the beneficial bacterium in the gut (prebiotics) is used to improve human health. Given that microbes release many metabolites, cell wall components, and proteinaceous molecules acting directly on the host tissues, these bacteria-derived factors mediate the beneficial or detrimental effect of the gut microbiome, collectively termed “postbiotics” (Aguilar-Toalá et al., 2018; Suez and Elinav, 2017). In the present study, a bacterial conditioning assay demonstrated the altered nutritional composition, as exemplified by the sharp reduction of purines within 24 hours of bacterial inoculation with *L. plantarum* Lsi. This fermentation of the diet can occur in natural laboratory conditions where the gut microbiome is synchronized to the dietary microbiome. We believe this assay can be used as a model for studying how postbiotics work on the host physiology. Indeed, there are some postbiotic mechanisms by which *Lactobacillus* spp. suppress uric acid in serum in mice on a high-purine diet (Li et al., 2014). It is interesting to test how much of the postbiotic effect is through reduction, rather than production, of a particular nutrient in the diet.

### Limitations of the Study

This study did not directly reveal how increased allantoin levels contribute to organismal aging. We tested allopurinol together with IMD activation in the Malpighian tubules, but failed to rescue the shortened lifespan. This observation was correlated with the accumulation of xanthine, which can also form stones and shorten lifespan. Also, neither the mechanism by which *A. persici* triggers IMD activation in aged animals nor how IMD regulates purine levels was elucidated.

### Resource Availability

#### Lead Contact

Further information and requests for resources and reagents should be directed to and will be fulfilled by the Lead Contact, Fumiaki Obata (fumiaki.obata@g.ecc.u-tokyo.ac.jp).

#### Materials Availability

All unique/stable reagents generated in this study are available from the Lead Contact without restriction.

#### Data and Code Availability

The original/source data are available from the Lead Contact on request. The result of 16S rRNA amplicon sequencing analysis has been deposited in DDBJ under the accession number DRA010501.

## Methods

All methods can be found in the accompanying Transparent Methods supplemental file.

## Supplemental Information

Document S1. Transparent Methods and Figures S1–S5
