# Supplementary material for: Gut Bacterial Species Distinctively Impact Host Purine Metabolites during Aging in Drosophila
Source: iScience. 2020 Sep 10;23(9):101477. doi: 10.1016/j.isci.2020.101477 (PMC7520893; doi:10.1016/j.isci.2020.101477)
Supplement: Document S1. Transparent Methods and Figures S1–S5 [file mmc1.pdf]

iScience, Volume *23*

Supplemental Information

Gut Bacterial Species Distinctively Impact Host Purine Metabolites during Aging in *Drosophila*

Toshitaka Yamauchi, Ayano Oi, Hina Kosakamoto, Yoriko Akuzawa-Tokita, Takumi Murakami, Hiroshi Mori, Masayuki Miura, and Fumiaki Obata

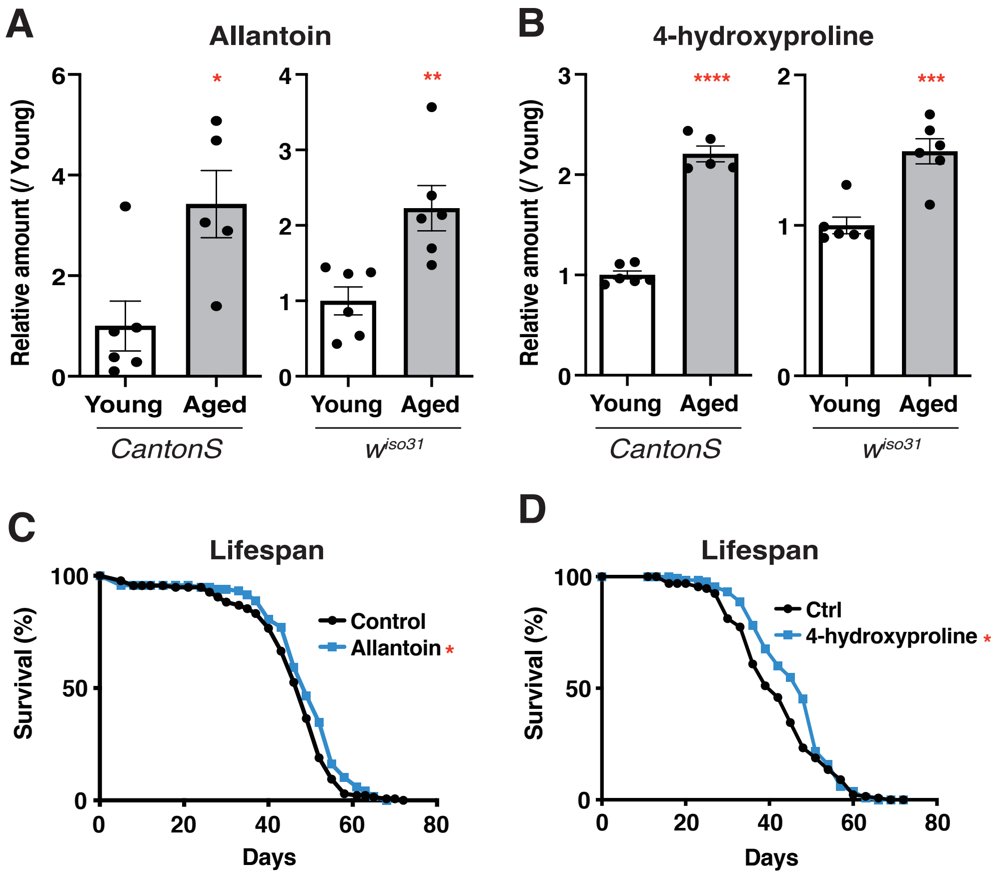

Figure S1. Allantoin and 4-hydroxyproline are robustly increased during aging, but they do not shorten lifespan. Related to Figure 1.

(A, B) Relative amount of allantoin or 4-hydroxyproline in whole body of aged (6-week-old) male flies (*CantonS, w^{iso31}*) compared to young (2-week-old) flies. n = 5–6. (C, D) Lifespan of w^{Dah} male flies fed with allantoin (1 mM) or 4-hydroxyproline (100 µg/mL). Concentration was determined based on the level of each metabolite in aged flies. n = 137 (Control), n = 118 (Allantoin) for (C). n = 133 (Control and 4-hydroxyproline) for (D). Log-rank test, $p < 0.05$. Data are represented as mean and SEM. Statistics, two-tailed Student's *t*-test. *, $p < 0.05$. **, $p < 0.01$. ***, $p < 0.001$. ****, $p < 0.0001$.

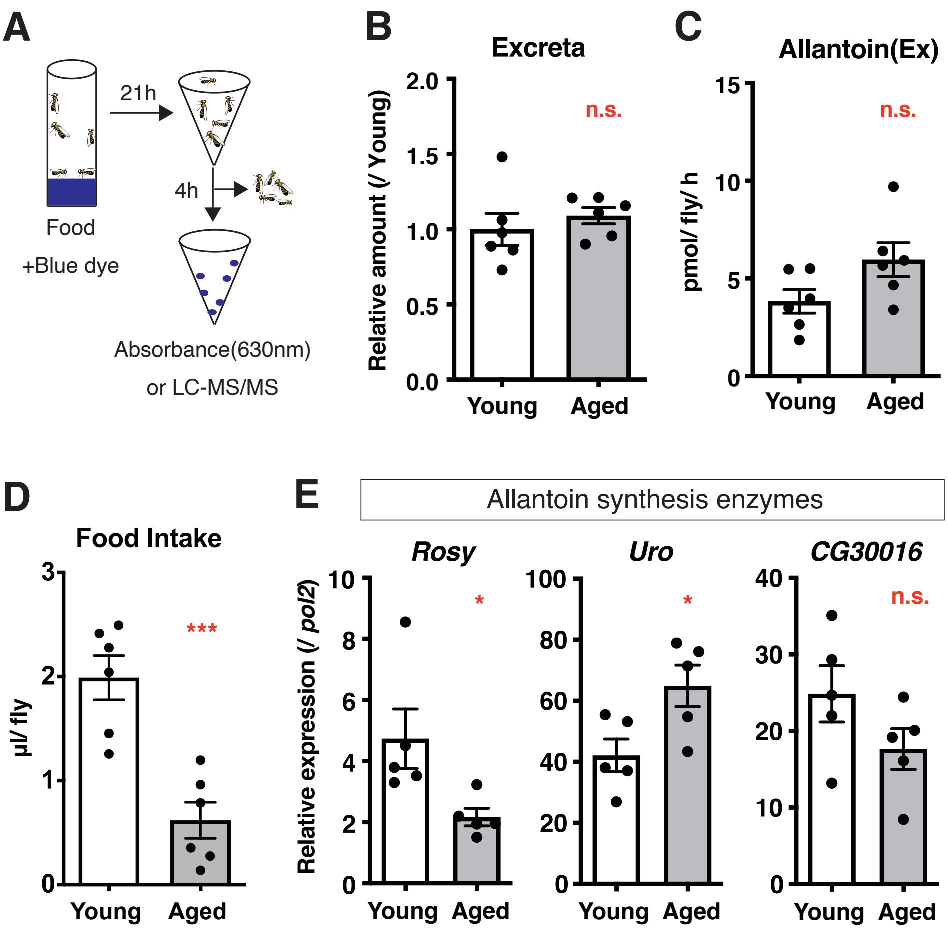

Figure S2. Quantification of excretion, food intake, and gene expression of enzymes for allantoin synthesis during aging. Related to Figure 2.

(A) Experimental scheme of excretion assay. (B) Amount of total excreta in aged (7-week-old) w^{Dah} male flies. Relative to young (1-week-old) w^{Dah} male flies. n = 6. (C) Quantification of allantoin by LC-MS/MS in excreta from young or aged w^{Dah} male flies. n = 6. (D) Quantification of food intake by the capillary feeder assay in young (2-week-old) and aged (5-week-old) w^{Dah} male flies. n = 6. (E) Quantitative RT-PCR of *Rosy, Urate oxidase* (*Uro*) *and CG30016* in the Malpighian tubules of young (1-week-old) or aged (7-week-old) w^{Dah} male flies. n = 5. Data are represented as mean and SEM. Statistics, two-tailed Student's *t*-test. *, $p < 0.05$. ***, $p < 0.001$.

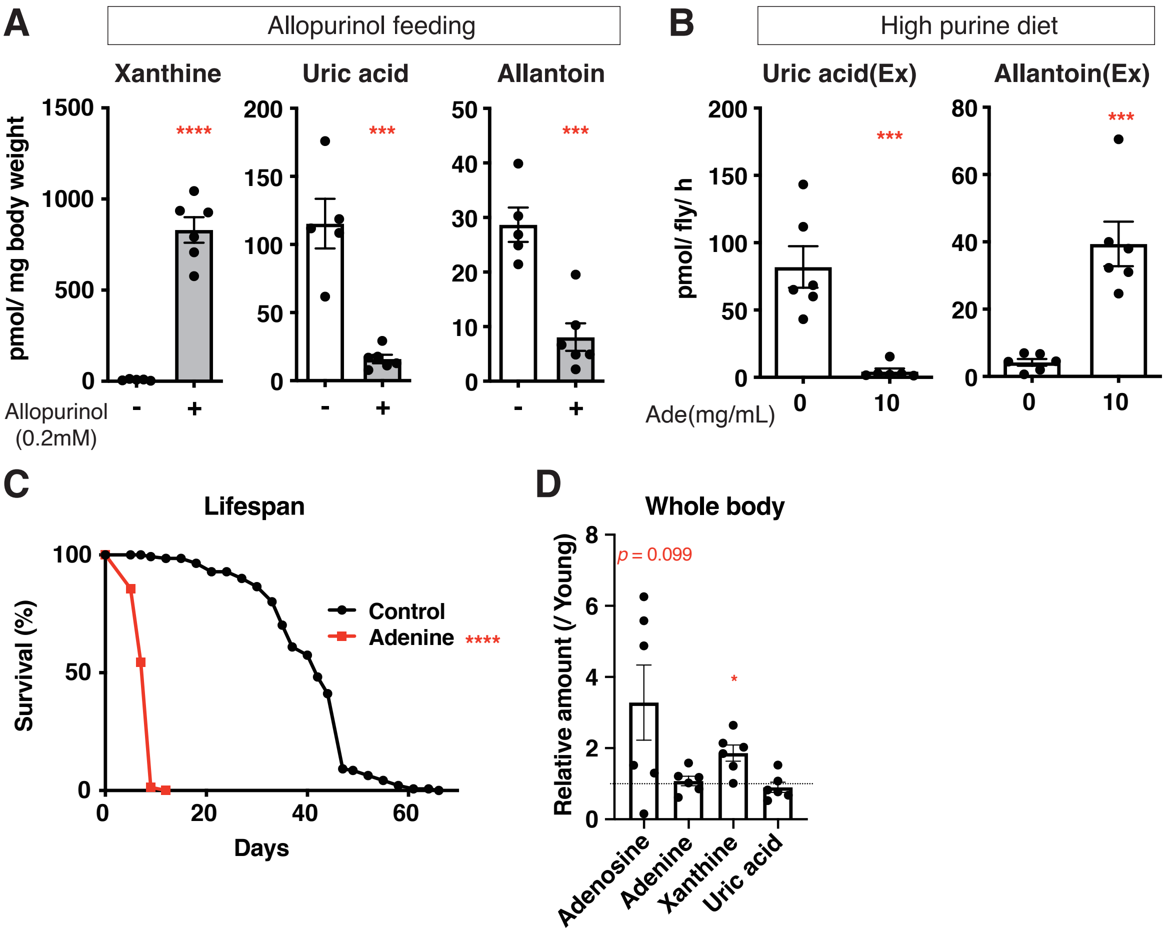

Figure S3. The amount of purine metabolites upon allopurinol, high-purine diet, or during aging. Related to Figure 2.

(A) Quantification of xanthine, uric acid and allantoin in whole body of young (1-week-old) w^{Dah} male flies fed with allopurinol (0.2 mM) for three days. n = 5–6. (B) Quantification of uric acid and allantoin by LC-MS/MS in excreta from young (2-week-old) w^{Dah} male flies fed with high-purine diet (Adenine, 10 mg/mL). n = 6. (C) Lifespan of w^{Dah} male flies fed with high-purine diet (Adenine, 10 mg/mL). n = 135 (Control), n = 147 (Adenine). Log-rank test, $p < 0.0001$. (D) Relative amount of adenosine, adenine, xanthine or uric acid by LC-MS/MS in whole body of aged (5-week-old) w^{Dah} male flies. Relative to young (1-week-old) flies. n = 6. Data are represented as mean and SEM. Statistics, two-tailed Student's *t*-test. *, $p < 0.05$. ***, $p < 0.001$. ****, $p < 0.0001$.

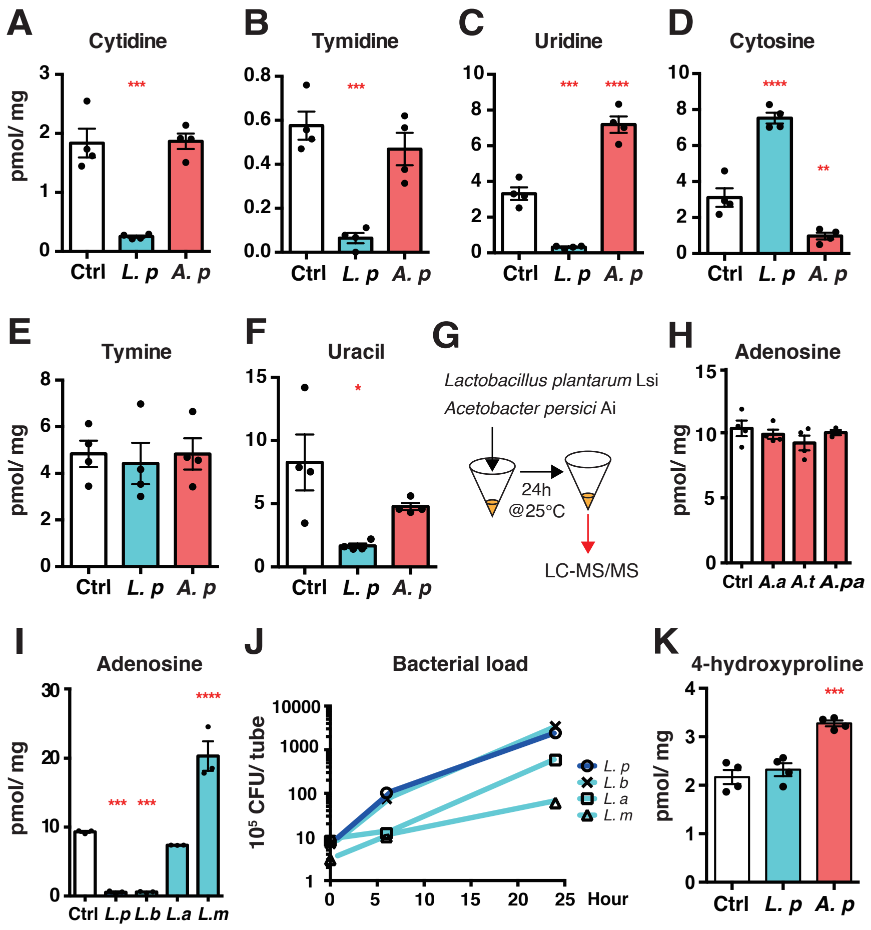

Figure S4. Bacteria modulate composition of fly diet in a species-specific manner. Related to Figure 4.

(A–F) Quantification of cytidine, tymidine, uridine, cytosine, tymine, and uracil by LC-MS/MS in fly diet conditioned with isolated bacteria strains. *L. p*, *L. plantarum* Lsi. *A. p*, *A. persici* Ai. Ctrl, control medium. n = 4. (G) Experimental scheme of bacterial conditioning assay in a 1.5 mL tube. (H, I) Quantification of adenosine by LC-MS/MS in fly diet conditioned with bacterial species. n = 4 for (H). *A.a*, *A. aceti* (NBRC 14818). *A.t*, *A. tropicalis* (NBRC 16470). *A.pa*, *A. pasteurianus* (NBRC 106471). n = 3 for (I). *L.p*, *L. plantarum* Lsi. *L.b*, *L. brevis* (NBRC 3345). *L.a*, *L. acidophilus* (NBRC 13951). *L.m*, *L. murinus* (NBRC 14221). (J) Colony forming unit (CFU) of four bacterial species (*L.p*, *L.b*, *L.a*, *L.m*) in fly diet. (K) Quantification of 4-hydroxyproline by LC-MS/MS in fly diet conditioned with isolated strains. n = 4. Data are represented as mean and SEM. Statistics, One-way ANOVA with Dunnett's multiple comparison test. *, $p < 0.05$, **, $p < 0.01$, ***, $p < 0.001$. ****, $p < 0.001$.

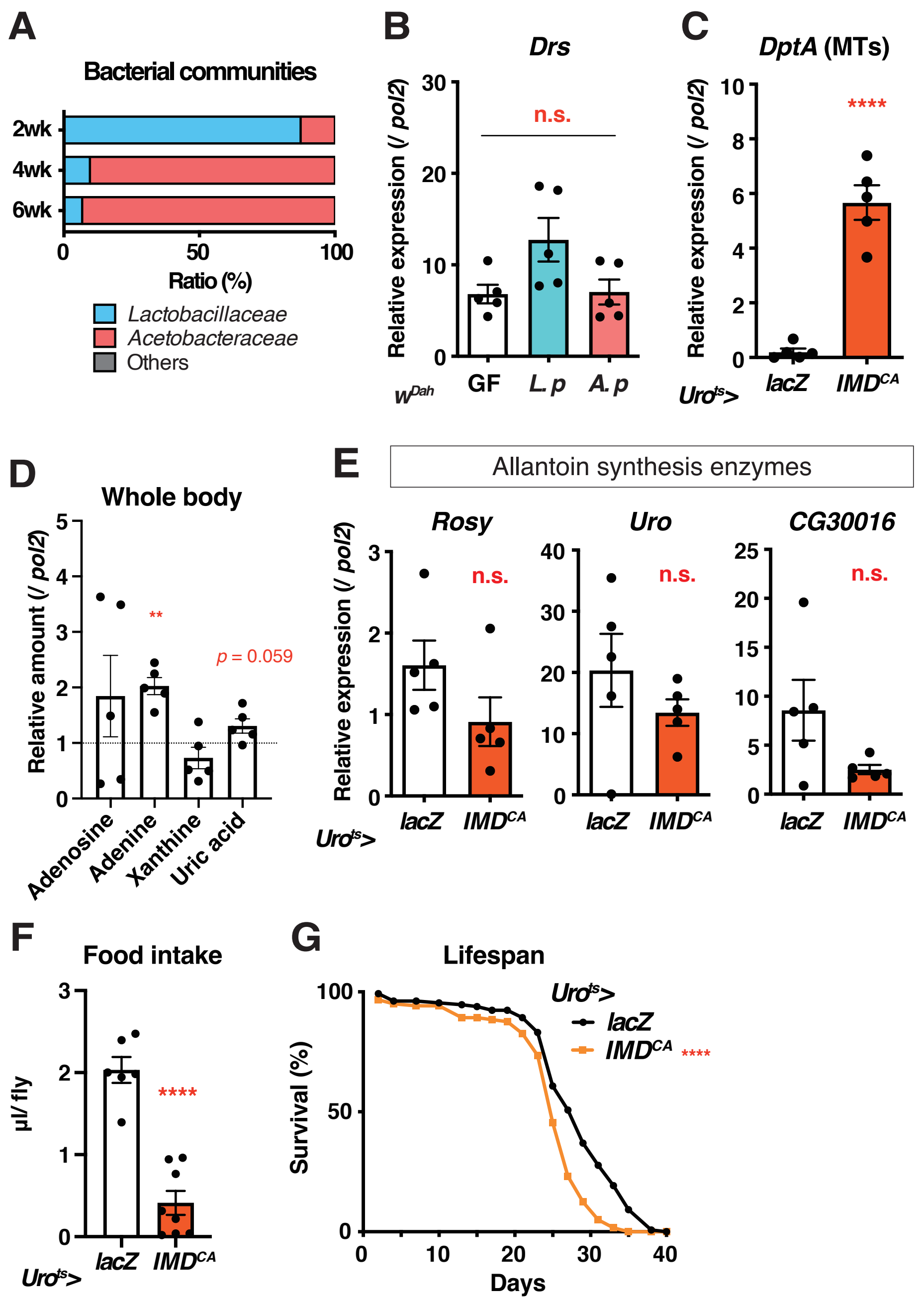

Figure S5. IMD activation, lifespan, and purine metabolism in the Malpighian tubules. Related to Figure 5 and 6.

(A) 16S rRNA amplicon sequencing analysis of gut microbiome in w^{iso31} flies. (B) Quantitative RT-PCR analysis of *Drosomycin* (*Drs*) in whole body of w^{Dah} young male flies mono-associated with the bacterial strains. *L. p*, *L. plantarum* Lsi. *A. p*, *A. persici* Ai. GF, Germ-free. n = 5. (C) Quantitative RT-PCR analysis of *Diptericin A* (*DptA*) of young male flies with ctrl (*lacZ*) or constitutive active form of IMD (*IMD*^{CA}) in the Malpighian tubules. n

= 5. (D) Relative amount of adenosine, adenine, xanthine or uric acid by LC-MS/MS in whole body of young (2-week-old) male flies fed with antibiotics. Either *lacZ* or IMD^{CA} was overexpressed in the Malpighian tubules. Relative to *lacZ* flies. $n = 5$. (E) Quantitative RT-PCR analysis of *Rosy, Urate oxidase* (*Uro*) *and CG30016* in the Malpighian tubules of young (2-week-old) male flies. Either *lacZ* or IMD^{CA} was overexpressed in the Malpighian tubules. $n = 5$. (F) Quantification of food intake by the capillary feeder assay in male flies. Either *lacZ* or IMD^{CA} was overexpressed in the Malpighian tubules. n = 6 (*lacZ*), n = 8 (IMD^{CA}). (G) Lifespan of male flies with overexpressing *lacZ* or IMD^{CA} in the Malpighian tubules. n = 130 (*lacZ*), n = 121 (IMD^{CA}). Log-rank test, $p < 0.0001$. Data are represented as mean and SEM. Statistics, One-way ANOVA with Dunnett's multiple comparison test (B) or two-tailed Student's *t*-test (C–F). ****, $p < 0.0001$.

Transparent Methods:

Fly husbandry and stock

Flies were reared on a standard diet containing 4 % cornmeal, 6 % baker's yeast (Saf Yeast), 6 % glucose (Wako, 042-31177), and 0.8 % agar (Kishida chemical, 260-01705) with 0.3 % propionic acid (Tokyo Chemical Industry, P0500) and 0.05 % nipagin (Wako, 132-02635). Flies were reared under 25 °C, 65 % humidity with 12 h/12 h light/dark cycles. The wild-type strains w^{Dah}, *Canton S* and w^{iso31} were used in the previous study (Obata et al., 2018). The fly lines were: *Da-GeneSwitch* (Dr. Veronique Monnier), *UAS-lacZ* (Dr. Goodman C), *UAS-IMD*^{CA} (Petkau et al., 2017), *NP1-Gal4, tub-Gal80*^{ts} (Jiang et al., 2009), *Uro-Gal4; tub-Gal80*^{ts} (Terhzaz et al., 2010), *Lpp-Gal4, tub-Gal80*^{ts}*/ TM6B* (Brankatschk and Eaton, 2010), *UAS-relish*-RNAi (BDSC: 33661), *UAS-Dredd*-RNAi (BDSC: 34070). *Da-GeneSwitch*, *UAS-lacZ* and *UAS-IMD*^{CA} are backcrossed with w^{iso31}. Adult flies eclosed within two days were collected and maintained for additional two days for maturation on standard diet. Then male flies were sorted with a density of 15 flies/ vial and flipped to fresh vials every three days. For lifespan analysis, the number of dead flies was counted every three days when flies were flipped to fresh vials.

Adult-specific genetic experiments were performed using *tub-Gal80*^{ts}. Flies were kept at 18 °C from embryo to adult flies and then shifted to 29 °C for six days or ten days. For GeneSwitch, RU486 (Wako, 84371-65-3) dissolved in ethanol (Wako, 052-00467) or the same amount of ethanol was added to standard diet to a final concentration of 200 μM, and flies were fed with the diet for six days.

Bacterial culture and strains

Isolated bacterial strains pre-cultured with MRS broth (Wako, 521-03485) for over night were cultured with the MRS broth for four to eight hours. Started OD_{600} was 0.01–0.1. Bacterial strains were: *L. plantarum* Lsi (Kosakamoto et al., 2020), *A. persici* Ai (Kosakamoto et al., 2020), *L. brevis* (NBRC3345), *L. acidophilus* (NBRC13951), *L. murinus* (NBRC14221), *A. aceti* (NBRC14818), *A. tropicalis* (NBRC16470), *A. pasteurianus* (NBRC106471). As for colony forming unit (CFU) count, obtained samples were scattered on MRS plate (OXOID, CM0361) and incubated at 30 °C for two days.

Dietary manipulations

High-purine diet was prepared by adding adenine hemisulfate (Sigma, A9126) to standard diet to a final concentration of two or ten mg/mL. For high-yeast feeding, male flies were fed with diet containing various concentrations of yeast extract (BD, 212750) in addition to 6 % glucose (Wako, 049-31165), 1 % agar, 0.3 % propionic acid, and 0.05 % nipagin for three days. For high-sugar feeding, male flies were fed with diet containing various concentrations of sucrose (Wako, 196-00015) in addition to 4 % yeast extract (BD, 212750), 1 % agar, 0.3 % propionic acid, and 0.05 % nipagin for three days. For allopurinol administration, male flies were fed with standard diet containing 0.2 mM allopurinol (Abcam, ab142565) for three days.

Bacterial manipulations

Antibiotics (200 µg/mL rifampicin, 50 µg/mL tetracycline, 500 µg/mL ampicillin) and 0.12 % nipagin were added to standard diet to remove all bacteria. To remove *Acetobacteraceae*, larvae were fed with 2.5 mM paraquat (1,1'-Dimethyl-4,4'-bipyridinium Dichloride, Tokyo Chemical Industry, D3685), and adult flies were collected in standard diet.

Germ-free flies were established by the bleach-based method. Embryos collected within four hours after egg laying were kept at 18 °C for overnight. The following day, larvae were carefully removed from collected embryos. Embryos were sterilized by 70 % ethanol three times and 3 % solution of sodium hypochlorites for five minutes. Embryos were then washed by sterilized water thoroughly. Embryos were transferred to vials containing antibiotics-supplemented diet (Ampicillin 50 µg/mL, Kanamycin 50 µg/mL, Tetracyclin 10 µg/mL, Erythromycin 10 µg/mL) for germ-free flies or vials containing UV-sterilized diet for mono-association of flies. For mono-association, 40 µL of bacterial culture (*L. plantarum* Lsi OD_{600} = 0.2, *A. persici* Ai OD_{600} = 0.02) was added directly on embryos. Gnotobiotic conditions of flies were constantly confirmed by plating on MRS agar.

Bacterial conditioning assay

For preparing bacterial-conditioned diet, *L. plantarum* or *A. persici*, 100 µL of cultured bacteria (OD_{600} = 0.2) was added on top of the standard diet (3 mL in fly vial) and incubated at 25 °C with 65 % humidity. Twenty-four hours later, the upper half of the conditioned diet

(30–50 mg) was collected and the composition was analysed by LC-MS/MS. For feeding flies with the bacterial-conditioned diet, 100 µL of the antibiotics-cocktail (10 mg/mL rifampicin, 2.5 mg/mL tetracycline and 25 mg/mL ampicillin) dissolved in 0.1N HCl was added to the bacterial-conditioned diet to inhibit/kill the proliferation of bacteria. We fed the flies with this diet for three days. For bacterial conditioning in a 1.5 mL tube, 2 µL of cultured bacteria (OD_{600} = 0.2) were added to the 30–50 mg of standard diet in a 1.5 mL tube. After 24 hours with 25 °C, 65 % humidity, the diet in the tube was analysed by LC-MS/MS or subjected to CFU count.

Quantitative RT-PCR analysis of immune activation or enzymes for purine metabolism

Total RNA was purified from four male flies or five to eight male Malpighian tubules using ReliaPrep RNA Tissue Miniprep kit (Promega, z6112). cDNA was prepared from 100–400 ng of the total RNA by the Takara PrimeScript RT Reagent Kit with gDNA Eraser (Takara bio, RR047). Quantitative PCR was performed using TB Green™ Premix Ex Taq™ (Tli RNaseH Plus, Takara bio, RR820W) and a Quantstudio 6 Flex Real Time PCR system (ThermoFisher) using *RNA pol2* as an internal control. Primer sequences were:

RNA pol2 forward, CCTTCAGGAGTACGGCTATCATCT.

RNA pol2 reverse, CCAGGAAGACCTGAGCATTAATCT.

Rosy forward, TGGTGACTTCCCACTGGAG.

Rosy reverse, GGTTCGGGTATTTCAAGCAG.

Uro forward, GCGATGTGGTTATAAGGAGAACA.

Uro reverse, TCTTCAGCACCCGGAGAC.

CG30016 forward, GATGCACGAAAGTTTTCTACCC.

CG30016 reverse, GGGATCTCCATTCCTGAATCT.

DptA forward, CGTCGCCTTACTTTGCTGC.

DptA reverse, CCCTGAAGATTGAGTGGGTACTG.

16S rRNA gene amplicon sequencing analysis

Flies were rinsed in 3 % bleach, 70 % ethanol and then washed extensively with PBS before dissection. Dissected guts (four guts per sample) were collected in PBS on ice, then

transferred to 270 µL lysis buffer (20 mM Tris pH8.0, 2 mM EDTA and 1% Triton X-100) with 20 mg/mL lysozyme from chicken egg (Sigma, L4919) and homogenized. Homogenized samples were stored at -80 °C. Frozen gut samples were thawed at 37 °C for 45 min in a 1.5 mL microcentrifuge tube, transferred to a 2 mL tube containing 0.1 mm glass beads (Scientific Industries, SI-BG01) and then incubated using a Mini-Beadbeater-24 (Biospec Products, 112011EUR) at 2,500 rpm for 20 s. After 15 min incubation at 37 °C, 30 µL of proteinase K and 200 µL Buffer TL (Qiagen) were added to each sample, followed by incubation at 56 °C for 15 min. Genomic DNA was purified by QIAamp DNA Micro kit (Qiagen, 56304), and V3–V4 variable region of 16S rRNA genes were amplified using 341f/806r primer set. PCR amplicons were purified using a QIAquick PCR Purification kit (Qiagen, 28104) and sent to Fasmac for Illumina MiSeq sequencing and analysis. The data were deposited to DDBJ (accession number, DRA 010501).

Genome analysis of *Lactobacilli*

Protein-coding sequences (CDSs) within the genomes of four *Lactobacillus* species were predicted and annotated by using DFAST pipeline with the Prodigal option (Hyatt et al., 2010; Tanizawa et al., 2018). In addition to the DFAST annotation, KEGG orthology identifiers were assigned to the CDSs by conducting BLASTP search against KEGG protein database (Kanehisa et al., 2014) with the following criteria: sequence identity ≥ 40 %, e-value < 1e-5, bit score ≥ 70, and aligned region covering 40 % and more of both query and subject sequences. Deduced CDSs among the four species were then clustered into homologous gene groups by using OrthoFinder v2.3.8 (Emms and Kelly, 2019).

Measurement of metabolites or diet composition

Liquid chromatography-tandem mass spectrometry (LC-MS/MS) was used to analyse fly metabolites and diet composition. Four or five whosle bodies of adult male flies or 30–50 mg of bacterial-conditioned diet were homogenized in 160 µL of 80 % methanol containing 10 µM of internal standards (methionine sulfone and 2-morpholinoethanesulfonic acid), then de-proteinised by 75 µL acetonitrile and 10 kDa column, followed by complete evaporation. The samples were resolubilised in water and injected to LC-MS/MS with PFPP column (Discovery HS F5 (2.1 mm x 150 mmL, 3 µm, Sigma-Aldrich) in the column oven at 40 °C. Gradient from solvent A (0.1 % formic acid, Water) to solvent B (0.1 % formic acid, acetonitrile) were

performed during 20 minutes of analysis. MRM methods for metabolite quantification were optimised using the software (Labsolutions, Shimazu). The data was normalized by methionine sulfone and wet weight of the sample. The Heat map and PLS-DA analyses were conducted by MetaboAnalyst 4.0 with the data after auto scaling (Chong et al., 2019).

Excretion assay

Flies were transferred onto standard diet containing 2 % blue dye (Brilliant blue FCF, Wako, 3844-45-9) at 25 °C, 65 % humidity for 21 hours. Five flies were then transferred to a 1.5 mL tube containing 20 µL of 1 % sucrose, 1 % agar and incubated for four hours. To collect excreta, 50 µL of water was added to the 1.5mL tube and dissolve by vortex, followed by measuring absorbance at 630 nm. To quantify metabolites by LC-MS/MS, excreta was collected by 300 µL of 80 % methanol containing 10 µM of internal standards (methionine sulfone and 2-morpholinoethanesulfonic acid). The samples were then subjected to the standard procedure for LC-MS/MS analysis as described above.

Purine or pyrimidine-depleted bacteria medium

We adapted a previously-reported chemically defined medium (Piper et al., 2017) for bacterial culture with following modifications. Cholesterol and agar were not included. To prevent precipitation, metal irons were reduced to one tenth of the original concentrations. For purine- or pyrimidine-depleted medium, inosine or uridine was depleted respectively. To assess the bacterial growth speed, bacteria precultured with purine/pyrimidine-depleted medium (OD_{600} = 0.02) were added in the corresponding medium and incubated at 30 °C for 21 hours.

Capillary feeder assay

Two glass capillaries containing 5 % yeast extract, 2 mg/mL red dye (Acid red 52, Wako, 3520-42-1), and n-Octyl acetate (1:100,000; TCI, 112-14-1) were inserted into the cap. Ten male flies were placed in each empty vial. The level of the food was marked, and the vials were laid in a container with wet towels to prevent water evaporation. The container was

incubated at 25 °C. After 24 hours, the amount of the food remained in the capillaries was recorded. The vial without flies was also included in the container to subtract evaporation.

Quantification and statistical analysis

Statistical analysis was performed using Graphpad Prism 8 except for survival curves where OASIS2 was used (Han et al., 2016). A two-tailed Student's *t*-test was used to test between two samples. One-way ANOVA with multiple comparison tests was used to compare among group. Statistical significance is; *, $p < 0.05$, **, $p < 0.01$, ***, $p < 0.001$, ****, $p < 0.0001$. Bar graphs were drawn as mean and SEM with all the data point shown by dots to allow readers to see the number of samples and each raw data.

Supplemental References:

Brankatschk, M., and Eaton, S. (2010). Lipoprotein particles cross the blood-brain barrier in Drosophila. J. Neurosci. *30*, 10441–10447.

Chong, J., Wishart, D.S., and Xia, J. (2019). Using MetaboAnalyst 4.0 for Comprehensive and Integrative Metabolomics Data Analysis. Curr. Protoc. Bioinforma. *68*, e86.

Emms, D.M., and Kelly, S. (2019). OrthoFinder: Phylogenetic orthology inference for comparative genomics. Genome Biol. *20*, 238.

Han, S.K., Lee, D., Lee, H., Kim, D., Son, H.G., Yang, J.S., Lee, S.J. V., and Kim, S. (2016). OASIS 2: Online application for survival analysis 2 with features for the analysis of maximal lifespan and healthspan in aging research. Oncotarget *7*, 56147.

Hyatt, D., Chen, G.L., LoCascio, P.F., Land, M.L., Larimer, F.W., and Hauser, L.J. (2010). Prodigal: Prokaryotic gene recognition and translation initiation site identification. BMC Bioinformatics *11*, 119.

Jiang, H., Patel, P.H., Kohlmaier, A., Grenley, M.O., McEwen, D.G., and Edgar, B.A. (2009). Cytokine/Jak/Stat Signaling Mediates Regeneration and Homeostasis in the Drosophila Midgut. Cell *137*, 1343–1355.

Kanehisa, M., Goto, S., Sato, Y., Kawashima, M., Furumichi, M., and Tanabe, M. (2014). Data, information, knowledge and principle: back to metabolism in KEGG. Nucleic Acids Res. *42*, D199–D205.

Kosakamoto, H., Yamauchi, T., Akuzawa-Tokita, Y., Nishimura, K., Soga, T., Murakami, T., Mori, H., Yamamoto, K., Miyazaki, R., Koto, A., et al. (2020). Local Necrotic Cells Trigger Systemic Immune Activation via Gut Microbiome Dysbiosis in Drosophila. Cell Rep. *32*, 107938.

Obata, F., Fons, C.O., and Gould, A.P. (2018). Early-life exposure to low-dose oxidants can increase longevity via microbiome remodelling in Drosophila. Nat. Commun. *9*, 975.

Petkau, K., Ferguson, M., Guntermann, S., and Foley, E. (2017). Constitutive Immune Activity Promotes Tumorigenesis in Drosophila Intestinal Progenitor Cells. Cell Rep. *20*, 1784–1793.

Piper, M.D.W., Soultoukis, G.A., Blanc, E., Mesaros, A., Herbert, S.L., Juricic, P., He, X., Atanassov, I., Salmonowicz, H., Yang, M., et al. (2017). Matching Dietary Amino Acid Balance to the In Silico-Translated Exome Optimizes Growth and Reproduction without Cost to Lifespan. Cell Metab. *25*, 610–621.

Tanizawa, Y., Fujisawa, T., and Nakamura, Y. (2018). DFAST: a flexible prokaryotic genome annotation pipeline for faster genome publication. Bioinformatics *34*, 1037–1039.

Terhzaz, S., Cabrero, P., Chintapalli, V.R., Davies, S.A., and Dow, J.A.T. (2010). Mislocalization of mitochondria and compromised renal function and oxidative stress resistance in Drosophila SesB mutants. Physiol. Genomics *41*, 33–41.
